# Neonatal intensive care unit nurses knowledge and attitude toward neonatal palliative care: review of the literature

**DOI:** 10.2144/fsoa-2022-0062

**Published:** 2023-04-11

**Authors:** Sawsan Abuhammad, Marah Elayyan, Hamza Ababneh

**Affiliations:** 1 Nursing Faculty, Jordan University of Science & Technology, Irbid, 22110, Jordan; 2 Pharmacy Faculty, Jordan University of Science & Technology, Irbid, 22110, Jordan

**Keywords:** attitude, knowledge, NPC, nurse

## Abstract

**Aim:**

To review studies regarding neonatal nurses' knowledge and attitude toward neonatal palliative care (NPC).

**Method:**

The researchers searched internet sources such as Google Scholar for NPC, Nurses, Knowledge, Attitude, and Educational Intervention.

**Results:**

Subheadings identified in the literature review were Nurses Knowledge toward NPC in NICU, Nurses Attitude toward NPC in NICU, correlation between Knowledge and Attitude toward NPC in NICU, The Effect of Educational program on Nurses Knowledge and Attitude toward NPC in NICU, and the Predictors of Knowledge and Attitude toward NPC among Nurses in NICU and Barriers to NPC provision and improvement.

**Conclusion:**

There are few studies from different nations regarding NPC found nurses have an inadequate knowledge of NPC, which also reflects their attitude.

## Plain language summary

**Objective:** To review studies regarding neonatal nurses' information and behavior toward end-of-life care. **Method:** The researcher searched internet sources such as Google Scholar, PubMed, Medline, and ResearchGate for end of life, Nurses, Information, Behavior, And Educational Program. **Results:** Subheadings identified in the literature review were nurses information toward end of life care in neonate care unit, nurses attitude toward end of life in neonate care unit, correlation between knowledge and attitude toward end of life in neonate care unit, the effect of educational program on nurses information and behavior toward end of life in neonate care and the predictors of information and behavior toward end of life among nurses in neonate and barriers to end of life. **Conclusion:** There are few studies from different nations regarding end of life found nurses have an inadequate information of end of life, which also reflects their behaviors.

It is critical to provide end-of-life care for neonates, families, and healthcare providers [1–3]. It is an enormous challenge filled with emotions and ambiguity, regardless of the need for end-of-life care for neonate, no strategy or information was provided regarding this type of care for neonates and their parents [4,5]. Parents of neonates around the time of death are feeling the loss of the neonate before their death, instead of being inspired with optimism and hope for a brighter future with their neonates [6]. Parents are anxious for their neonates that may suffer from anguish and physical pain at the time of end-of-life. They wish their neonate's death to be as comfortable as possible. The NPC seeks to avoid and relieve physical, emotional and psychological pain in sick neonates [7]. Healthcare teams that include nurses should focus on pain and symptom management as well as patient support anywhere at any given time, there are many unknown factors. Healthcare providers that include nurses and families are undergoing adjustments [8]. It is hard to forecast how long somebody will live, whether in hours, minutes, days, or weeks [9]. Despite all the challenges, more research into the attitudes and demands of Jordanian NICU nurses for NPC is needed to better our awareness of the problem and develop appropriate NPC guidelines for use in Jordanian hospitals.

Nurses require education as well as practical knowledge in the assessment and management of neonates' symptoms, helping and giving experienced care at the time of death [10]. Nurses have been at the forefront of initiatives to change organizational policies and cultures to promote NPC of critically ill neonates [9]. In Taiwan, Lee *et al.* [11] compared neonatologists' and neonatal nurses' schooling requirements in terms of NPC. According to the findings, 50% of neonatal nurses needed further pain management education. The need to improve the quality of NPC is becoming more widely recognized [7]. There are now several models in place where neonates and their families can get care focused on quality of life, peaceful dying, and family support. Previous work has identified nursing education needs that would prepare nurses to participate in NPC as part of their everyday routine in the NICU [12]. The available standards for knowledge of NPC among NICU nurses in Jordan are those provided by the selling company [8], which typically include instructions on how to operate the machines, how to select the proper size of cannulas for patients, how to rinse out, and how to control flow rates. Furthermore, earlier data revealed that there is a deficiency in nursing knowledge and practice for NPC among NICU nurses. The goal of this study is to review studies regarding neonatal nurses' knowledge and attitude toward NPC and the effect of educational intervention on nurses' knowledge and attitudes toward NPC in NICU.

## Search strategy

The researcher searched internet sources such as Google Scholar, PubMed, Medline, CINAHL, Proquest, Wiley and ResearchGate that university has an access over them for keywords such as NPC, Nurses, Knowledge, Attitude, And Educational Intervention. The authors also conducted a manual comprehensive online examination of reference lists from similar papers. Full-text English language papers published between 2010 and 2022 were included in the study since these studies reflect most current studies in the field. This search included studies that were comparative, cross-sectional descriptive, observational, surveys, and interventional studies. A total of 66 papers matched the eligibility requirements, and 40 full-text articles were reviewed and reported. The articles that were eliminated did not discuss NPC or looked at professions other than nursing.

Some of subheadings identified in the literature review were Nurses Knowledge toward NPC in NICU, Nurses Attitude toward NPC in NICU, Correlation between Knowledge and Attitude toward NPC in NICU, The Effect of Educational program on Nurses Knowledge and Attitude toward NPC in NICU, the Predictors of Knowledge and Attitude toward NPC among Nurses in NICU, and Barriers to NPC provision and improvement.

## Knowledge toward NPC among nurses in NICU

Another subheading emerged from the literature was Nurses Knowledge toward NPC among Nurses in NICU [11,13–17]. All of these studies showed that NICU nurses suffered from poor knowledge regarding NPC. For example, in the Australian NICU, Ahern [13] supports that NICU nurses need knowledge about the care of the dying neonate as nurses and knowledge about ethnicity and grief of the dying neonates. Also, practitioners place significantly higher priority on topics related to basic skills such as knowing the right words to say, managing symptoms, and documentation. Similarly, Gallagher [14], found that 93% of those participating reported they had poor knowledge about NPC or end-of-life care, and the study suggested that patients and their families should be educated about legal and clinical difficulties for NPC.

Gu *et al.* [15] found that most of the nurses in the study had no knowledge of any NPC policies or guidelines in their unit. In a different approach, Kilcullen and Ireland [16] in a qualitative study conducted in Australia among eight neonatal nurses, the results showed that nurses recognize the necessity of clinical knowledge, especially NPC education and the ability to adapt and modify care for families, these nurses emphasized the importance of robust clinical guidance, communication, and assessment of palliative care.

Two studies conducted in Taiwan regarding NPC among nurses in NICU [11,17]. These two studies used cross-sectional study designs. The results showed neonatologists (70%) showed deficiency in knowledge about CPR with families and helping with ethical decision-making as many other nurses were deficient in knowledge regarding pain management.

In summary, the literature showed that the nurses in NICU have some knowledge deficit regarding NPC. This means nurses and healthcare providers need more training and education to increase their knowledge about NPC.

## Nurses attitude toward NPC among nurses in NICU

Another subheading emerged from the literature was nurses' attitude toward NPC among nurses in NICU [18–23]. All the studies were showed that nurses had negative attitude toward NPC before applying and educational NPC programs. Chatziioannidis [18] found a statistically significant association was observed between shift work and nurses' attitudes toward NPC in NICU. Similarly, Chin *et al.* [19] found that nurses working in a unit with NPC policy and who had received palliative care education demonstrated more positive attitudes toward NPC than other nurses.

Two studies were conducted in Iran [20,22]. The authors found the nurses' attitude toward NPC was positive. A statistically significant association was observed between shift work and nurses' attitudes toward NPC. It is necessary to reinforce their positive attitude by providing clinical and theoretical training. Similarly, Mohammadi [22] found that most healthcare professionals had a positive attitude in involving caregivers with NPC in NICU.

In China, Gu *et al.* [15] showed that participating neonatologists experienced greater stress and failure in NPC than nurses, which might be linked to neonatologists' more positive attitudes regarding the relevance of palliative care and NPC education as compared with nurses. On the other hand, Kyc [21] found that the medical staff had significantly more negative attitudes regarding multiple resource-related items: assistance from peers, availability of counseling, and ability to spend time with families of dying neonates. In Jordan, Raziq [23] found all nurses (96%) had a negative attitude about giving drugs for the purpose of ending the life of the neonate, and 63% continuing the current treatment without adding other drugs. The author perceived impact of nurses' end-of-life decisions on their daily lives, and the importance of religious values in the nurses' personal lives.

In summary, all nurses have a positive attitude to providing palliative care, and it is necessary to strengthen their positive attitude by providing clinical and theoretical training. It is also important to pay more attention to eliminating other factors that increase their negative attitude toward NPC and implementation of palliative care.

## Correlation between knowledge & attitude toward NPC among nurses in NICU

Another subheading was emerged from the literature is correlation between knowledge and attitude toward NPC [8,19,24–30]. These all studies support the correlation between knowledge and attitude toward NPC among NICU nurses. For example, in Arabic countries three studies were conducted regarding correlation between knowledge and attitude toward NPC, Abudari *et al.* [24] in Saudi Arabia found there is a significant correlation between knowledge and attitudes toward NPC. Nurses who received NPC training had more favorable attitudes toward NPC. Similarly, Abuhammad and Almasri [8] found a significant positive correlation between the baseline knowledge and baseline attitude. Also, the knowledge correlated with the attitude at baseline the magnitude of the relation was higher post the intervention, and the attitude at baseline correlated with the post attitude.

In Egypt, Sabaq and Khalaf [28] found a statistically significant correlation between knowledge and practice scores of the staff nurses. Also, there is a positive correlation between knowledge and attitude with years of experience. In the USA, Chen *et al.* [19] found that nurses working in a unit with the NPC policy and who had received palliative care education demonstrated more positive attitudes toward NPC. Similarly, Wi in South Korea, found comfort level regarding attitude toward palliative care was positively correlated with knowledge and the perception of death. Nurses' role showed a positive correlation with perception of death.

Two experimental studies were conducted for the correlation between knowledge and attitude toward NPC among nurses in NICU [25,29]. The authors found that the educational intervention resulted in a substantial increase in knowledge of services, confidence in referring to these services as well as a reported change in attitudes toward NPC. Karkada and Nayak [26] found that there is a negative correlation between nurses' knowledge and attitude scores toward NPC. Similarly, Nepal *et al.* [27] found a negligible correlation between the level of knowledge and attitude. Ethnicity and religion were statistically significant with the level of knowledge. Also, Icare for dying relatives was statistically significant with the attitude toward NPC. In summary, the studies showed that nurses had negative attitude toward NPC. Improving the nurses' knowledge of NPC has a positive effect on improving the nurses' attitude.

## The effect of educational intervention on nurses knowledge & attitude toward NPC among nurses in NICU

Another subheading emerged from the literature was the effect of educational intervention on NICU nurses' knowledge and attitude toward NPC [19,28,29,31–37]. Many studies suggested positive impact of education program on knowledge and attitude toward NPC. For example, Bry *et al.* [38] analyzed the impact of a course in communication about NPC consisting of a two-hour interactive lecture and one-day hands-on workshop on the content of nurses' encounters with parents and the nurses' attitude to the empathetic needs of parents in a level III NICU. Similarly. Chen *et al.* [19] found that nurses working in a unit with the NPC policy and who had received palliative care education demonstrated more positive attitudes toward NPC. Educational policies and programs are important strategies to promote high-quality care for high-risk neonates and their families.

Hall *et al.* [32] and Murakami *et al.* [35] found there was a significant improvement in knowledge and attitudes after taking the course and 6-month follow-up. These results suggested that the participants' memory and learning strategy reinforced a positive attitude to end of life care. Similarly, four pre-post-tests studies were conducted regarding the impact of educational programs on NPC knowledge and attitude among nurses [29,33,36,39]. The authors found that post-test NPCQN knowledge and attitudes of NPC improved significantly after the intervention, indicating that educating nurse practitioners about EOL is an effective mechanism for increasing knowledge and improving attitudes toward NPC. Similarly, Twamley [29] found that a short (half-day) workshop can change attitudes and increase knowledge of the neonatal staff regarding NPC. Analysis of the open and closed text responses revealed a shift in attitude after the sessions from a moribund/end-of-life focus, to integrating palliative care as part of an overall treatment plan. Zhang and Lane [36] found comfort level significantly improved after interventions and no statistically significant change in participation scores.

In Egypt, Sabaq and Khalaf [28] found statistically significant improvements in many areas related to knowledge and attitudes toward NPC, such as symptom management, family support and comfort care. Similarly, Samsel *et al.* [34] found an increase in reorientation of care and use of palliative medications and a decrease in the diversity of use of end-of-life interventions, implementation of the NPC initiative was associated with an increase in palliative interventions for neonates in the last 48 h of life, suggesting that such an initiative might enhance the knowledge and attitude of nurses toward end-of-life care.

In summary, the literature showed that education programs enhance knowledge and attitude toward NPC. Educating professional nurses about NPC is an effective technique for both improving knowledge and changing attitudes, according to research on education among NICU nurses. More education is also recommended to enhance knowledge and attitude toward NPC.

## The predictors of knowledge & attitude toward NPC among nurses in NICU

Another subheading emerged from the literature was the predictors of knowledge and attitude toward NPC [18,23,39–42]. Azzizdeh *et al.* [40] revealed some correlations between several of the participants' core characteristics and NiPCAS scores. The nurses, who had a low level of education such as diploma or associate degree and who had no formal education program such as MNCY SCN neonatal palliative care program about NPC and NewYork-Presbyterian Neonatal Comfort Care Program had a negative attitude toward NPC. Also, the results indicated that there was a relationship between the age of nurses and their attitude [39] toward NPC. Chatziioannidis [18] found that the most important predictors of nurses' attitudes were paternity, involvement in daily practice, and supportive of current legislation reform. Similarly, Ismail *et al.* [42] found there was a correlation between affect gender, educational degree and knowledge and attitude toward NPC. Whereas religion was not a predictor of knowledge and attitude toward NPC. In Osehra and Mager [39] study found a correlation between age and the attitude of personal spirituality, as age increased, the attitude toward NPC was enhanced. Moreover, the experience in the nursing profession demonstrated a moderate linear effect on nurses' attitude of NPC. Also, the finding showed that nurses who actively practiced their religion scored significantly higher in attitude toward NPC than those who did not have faith. In Jordan, Raziq [23] reported that religion had a positive effect on nurses' attitude of NPC.

Regarding the relation between characteristics of the neonatal nurses and their knowledge and attitude toward NPC, there was a significantly higher effect of gender, religion, culture, and professional degree on knowledge and attitude toward NPC.

## Barriers to NPC among NICU nurses

Some studies emerged from the literature with the subheading regarding the barriers to providing NPC and improvement [15,16,19–21,40,43–46] Firstly, Azzizadeh *et al.* [40] found that 42.63% of the nurses agreed there are many barriers for providing NPC in NICU proposed by the National People's Congress. These barriers were ‘Inadequate resources’, and ‘Inappropriate personal and social attitudes’ scored the highest and lowest scores for all groups, respectively. Cortezzo *et al.* [47] and Ghazanchaie *et al.* [20] found that barriers to providing NPC were the presence of insufficient resources, improper application of technology, organizational culture, and professional competence of nurses. These barriers were classified as intermediate barriers. Similarly, Gibson *et al.* [48] found that the identified barriers for nurses to providing neonatal end-of-life care were perceived lack of self-assurance in the provision of care, team communication issues, and shared-decision making conflicts. Gu *et al.* [15] found five barriers for providing NPC: (1) discomfort with the use of technological life support; (2) parental demands; (3) a feeling of personal failure when a neonate dies; (4) a feeling of personal trauma when caring for dying neonates; and (5) the belief that curative care in the NICU is far more essential than NPC.

Three qualitative studies were conducted regarding perception of nurses of barriers of providing NPC among nurses [16,44]. In Australia, Kain and Victoria [11], Kidd *et al.* [49] and Kilcullen and Ireland [16]. They have found that staff perceptions of education, lack of confidentiality, isolation, staff attributes, and other systemic factors all have an impact on NPC delivery. Similarly, Kyc *et al.* [21] have identified three barriers to NPC, including (1) physical environment, (2) technical requirements, and (3) the societal factor. Finally, Wright *et al.* [46] determine five barriers of NPC: lack of formal education; pressure of parental demands; physical environment being unconducive for palliative care; nurses' inability to express opinions; and technological discrepancies.

In summary, the studies found that many barriers may impact the ability in providing NPC such as lack of education, negative attitude, pressure of parental demands; physical environment being unconducive for palliative care; nurses' inability to express opinions; and technological discrepancies.

## Conclusion

In conclusion, this literature review the studies that focused on knowledge and attitude toward NPC among nurse in NICU. This review included a variety of qualitative, systematic review, descriptive and cross-sectional approaches. It was the same result reached by researchers: nurses have an inadequate knowledge of NPC, which also reflects their attitude. Moreover, educational intervention on nurses knowledge would improve their knowledge and attitudes toward NPC and NICU.

## Future perspective

Studies of managing symptoms and pain among neonate are of great importance. It is necessary to confront misunderstandings regarding the application of opiates, such as the lack of morphine use in the diagnosis of dyspnea and pain in the end stages; how to continue improving physical effects in the lack of some traditional treatments among neonate; and how to teach emotional support in Jordan, where the term ‘religious’ is not generally recognized or used. Given the challenges and obstacles in engaging in research and publishing papers in English, we should emphasize the relevance of research in the future and develop our research skills based on clinical experience. We might also think about collaborating with scholars from wealthy nations and areas who have done similar work. Hospice and palliative care studies among neonate. The NPC research should be a worldwide effort since we all have a lot to offer and learn from one another.

**Summary points**

**Background**

- It is critical to provide end-of-life care for neonates, families, and healthcare providers.

**Findings**

- There is a deficiency in knowledge regarding NPC among NICU nurses.
- The educational programs have positive on knowledge and attitude toward NPC among NICU nurses.
- Many other factors such as experience and educational level that impact NPC among nurses in NICU.

**Future perspective**

- It is important to emphasize the relevance of research in the future and develop our research skills based on clinical experience.
